# Exploring the causal link among statin drugs and the osteoarthritis risk based on Mendelian randomization research

**DOI:** 10.3389/fgene.2024.1390387

**Published:** 2024-09-04

**Authors:** Wenjie Chen, Zhi Sun, Xinhai Xiong, Haitao Tan, Junhao Hu, Chenrui Liu, Cheng Chen

**Affiliations:** ^1^ 926 Hospital of the People's Liberation Army (Kunming University of Science and Technology Affiliated Hospital), Kaiyuan, China; ^2^ 921 Hospital of the People's Liberation Army (The Second Affiliated Hospital of Hunan Normal University), Changsha, China

**Keywords:** Mendelian randomization, statins, osteoarthritis, causality, compliance

## Abstract

**Purpose:**

Statins may have a protective effect against osteoarthritis (including knee osteoarthritis and hip osteoarthritis); however, the link between statins and osteoarthritis risk is incompletely established. The aim of this study was to explore the relationship between statins and osteoarthritis risk through Mendelian randomization analysis using pooled information from a large population-wide genome-wide association study (GWAS).

**Methods:**

Statin-related single-nucleotide polymorphisms (SNPs) were obtained from FinnGen’s latest 9th edition database, and data on osteoarthritis, knee osteoarthritis, and hip osteoarthritis were acquired from the IEU OpenGWAS, the UK Biobank, and Arthritis Research UK Osteoarthritis Genetics (ArcOGEN) database, respectively. The inverse-variance weighting method is an important analysis method to estimate the causal effect. Weighted median method, simple median method, weighted median estimator method, and MR–Egger regression were employed to supplement the explanation. Odds ratio and 95%CI were used to evaluate the causal relationship among statins and the osteoarthritis risk, osteoarthritis in the knee, and osteoarthritis in the hip. Second, sensitivity analysis was carried out to validate the reliability of the results. Cochran’s Q test was employed to test heterogeneity, MR–Egger intercept was employed to test whether horizontal pleiotropy existed, and single-nucleotide polymorphisms with potential influence were determined by the one-method analysis.

**Results:**

(1) The results of the inverse variance weighting method showed Gene prediction indicated that statins were associated with osteoarthritis (OR = 0.998, 95% CI: 0.996–0.999, P = 0.01) and knee osteoarthritis (OR = 0.964, 95% CI: knee (0.940–0.989, P = 0.005) and hip osteoarthritis risk were associated (OR = 0.928, 95% CI: 0.901–0.955, P = 4.28 × 10^−7^). (2) MR–Egger intercept analysis did not detect potential horizontal pleiotropy (osteoarthritis: P = 0.658; knee osteoarthritis: P = 0.600; and hip osteoarthritis: P = 0.141). (3) The findings provide evidence that statins reduce osteoarthritis risk, osteoarthritis in the knee, and osteoarthritis in the hip, as described in observational studies, and the specific mechanisms by which statins treat osteoarthritis require further investigation.

**Conclusion:**

The results of this study, at the genetic level, reveal a negative causal relationship between statins and osteoarthritis, and this causal relationship is also present in knee and hip osteoarthritis. This study provides evidence against the potential of statins in the treatment of osteoarthritis, prompting the clinical treatment of osteoarthritis to consider improving the start and compliance of statins in the future.

## Introduction

Osteoarthritis is a long-term, prominent degenerative disease in joints that occurs primarily in the hip and knee, including knee osteoarthritis and hip osteoarthritis, and is distinguished by degeneration in articular cartilage, long-term pain, deformities in joints, and eventually disability (Zhang et al., 2022; Perry et al., 2021). According to an epidemiological study, osteoarthritis affects over 500 million people globally, representing 7% of the world’s population, and the prevalence of the disease is increasing (Hunter et al., 2020). A wide range of metabolic abnormalities like diabetes, obesity, insulin resistance, and dyslipidemia are strongly associated with osteoarthritis, and these abnormalities also increase the cardiovascular disease risk (Hunter et al., 2020; Dancevic and McCulloch, 2014). Therefore, osteoarthritis is not just a disease related to age or body mass but a disease related to metabolic syndrome, where low levels of systemic inflammation stem from the effects of metabolic abnormalities. Inflammation and abnormal metabolic status can lead to degeneration in cartilage and the progression of disease (Dancevic and McCulloch, 2014; Papathanasiou et al., 2021). Currently, the goal of osteoarthritis treatment is to control pain and enhance function in joints. Nonsteroidal anti-inflammatory drugs, analgesics, and topical corticosteroids are commonly used drug treatment options (Sellam et al., 2020). Unfortunately, however, there is a lack of drugs that can alter the progression of osteoarthritis, which results in further cartilage damage progression that may eventually require surgical intervention.

Statins are rate-limiting enzymes involved in the synthesis of cholesterol that suppress 3-hydroxy-3-methylglutaryl-CoA reductase and have been shown to be effective in decreasing the prevalence and mortality of cardiovascular disease (Cai et al., 2021; Deligiorgi et al., 2021). Nowadays, the effects of statins on osteoarthritis have been of great interest to researchers as they can be used to prevent cartilage degeneration and improve osteoarthritis progression through anti-inflammatory, immunomodulatory, and lipid-lowering properties (Veronese et al., 2019; Saberianpour et al., 2022). One study showed that statin users had a 40% lower incidence of clinical osteoarthritis than non-users (Kadam et al., 2013). Additionally, there was a dose-dependent decrease in the occurrence of degenerative joint disease in the spinal cord among the comparisons of statin users with non-users (Cheng et al., 2018). However, there is debate about whether the effect of statins on reducing the risk of osteoarthritis is effective or increases the risk, which may be due to variations in inclusion criteria, study design, participant characteristics, statistical methods, and the assessment of end points (Haj-Mirzaian et al., 2019; Wang et al., 2020). Randomized controlled trials are the gold standard method for assessing causality, but they are difficult to conduct due to ethical and practical constraints.

In recent years, with the fast growth in the field of genetics, the Mendelian randomization (MR) method has been widely used in medical research (Gu et al., 2023). This method is a statistical model that uses single-nucleotide polymorphisms (SNPs) with the strong correlation of exposure factors as an instrumental variable to infer the causal relationship among susceptibility and study results (Yin et al., 2022; Chen et al., 2022). Traditional observational studies often face the challenges of confusion bias and reverse causality, which limit their ability to infer causality. By using instrumental variables as interventional variables, the MR method has become an effective tool to evaluate the interrelationship among susceptibility factors and disease onset due to its unique methodological characteristics and the advantages of less confusion and bias interference so that MR has a wide range of application prospects in disease prevention, treatment, and public health policymaking (Xu et al., 2022; Ho et al., 2022). Therefore, the two-sample MR analysis method was adopted in this study to probe the causal interrelationship between statins and osteoarthritis (including knee and hip osteoarthritis) and reveal the mechanism of the occurrence and progression of osteoarthritis through genetic analysis, providing a new basis for strengthening prevention and treatment measures.

## Materials and methods

### Research design

The study was based on the reports of enhanced observational epidemiological studies using MR (STROBE-MR) guidelines. In this two-sample MR study, the genome-wide association study (GWAS) database provided published single-nucleotide polymorphism sites as instrumental variables to determine the effects of statins on osteoarthritis risks, osteoarthritis in the knee and hip. For MR analysis, the inverse-variance weighting (IVW) method was employed to understand the causal effect between susceptibility and results. MR studies need to satisfy three key assumptions: first, the single-nucleotide polymorphisms selected should be linked to a significant exposure (statins). Second, SNPs must be independent to potential confounders between susceptibility and results; and third, SNPs were not directly associated with outcomes (osteoarthritis, knee osteoarthritis, and hip osteoarthritis) and could only be causally linked to statins.

### Exposed data

Statin GWAS-pooled statistics from the FinnGen study’s latest edition 9 biobank used self-reported data on routine medication and divided drugs into 23 classes. These categories are based on the Anatomical Therapeutic Chemistry (ATC) classification system developed by the World Health Organization, according to therapeutic, pharmacological, and chemical properties, and include up to 218,792 subjects of European descent (68,782 cases and 15,010 controls).

### Outcome data

The primary outcome of the study was clinically diagnosed osteoarthritis based on clinical conditions at a diseased level that required replacement of joints or based on the radiological evidence of disease (Kellgren–Lawrence scale ≥2). Genetic data for osteoarthritis came from the IEU OpenGWAS database at the MRC Institute for Integrative Epidemiology, which primarily contains publicly available aggregate GWAS data. GWAS for osteoarthritis were searched in the data, taking into account the sample size, sequencing depth, ethnicity, and data update time. The study selected Hawker’s published genome-wide osteoarthritis genetic dataset, which included 462,933 European subjects (38,472 cases and 424,461 controls).

In addition, knee osteoarthritis and hip osteoarthritis were also included in the study outcomes, given that both hip and knee joints are clinically major sites of osteoarthritis. The study used pooled-level information from the UK Biobank and Arthritis Research UK Osteoarthritis Genetics (ArcOGEN) database of the European ancestry cohort. Pooled-level data for knee osteoarthritis included 403,124 European subjects (24,955 cases and 378,169 controls). Pooled-level data for hip osteoarthritis included 393,873 European subjects (15,704 cases and 378,169 controls). The aforementioned information is derived exclusively from published research or publicly accessible GWAS data that have been granted ethical approval and informed consent and does not necessitate independent ethical approval. Details of the data are shown in Table 1.

**TABLE 1:** Genome-wide association analysis data from Mendelian randomization studies.

Exposure or outcome	Data source	Genome-wide association analysis data id number	Sample race	Number of single-nucleotide polymorphism	Sample size	Data source alliance	Data publication year
Statins	https://www.finngen.fi/en/access_results	finn-b-RX_STATIN	Europe	16,380,466	218,792	FinnGen biobank	2021
Osteoarthritis	https://gwas.mrcieu.ac.uk/	ukb-b-14486	Europe	9,851,867	462,933	MRC-IEU	2018
Knee osteoarthritis	http://www.arcogen.org.uk/	ebi-a-GCST007090	Europe	29,999,696	403,124	ArcOGEN	2019
Hip osteoarthritis	http://www.arcogen.org.uk/	ebi-a-GCST007091	Europe	29,771,219	393,873	ArcOGEN	2019

### Instrumental variables

First, the single-nucleotide polymorphisms selected for MR analysis must be nearly associated with exposure. In this study, single-nucleotide polymorphisms with significant genome-wide variations (P < 5 × 10^−8^) were selected, and their connection disequilibria (r2 = 0.001 and kb = 5,000) were tested as tools. The single-nucleotide polymorphisms of linkage imbalance are then eliminated, thus ensuring that the independence of the instrumental variables is satisfied. Second, in order to satisfy the hypothesis, the next analysis was conducted after taking confounders into account, and the PhenoScanner database was used to manually screen and delete the single-nucleotide polymorphisms associated with confounders and osteoarthritis results. Finally, according to the assumptions of the MR analysis, the F-statistic of a single-nucleotide polymorphism was measured to assess the strength of the selected single-nucleotide polymorphisms in order to ensure a robust association between the instrumental variable and exposure. F-statistic greater than 10 indicates a low likelihood of weak instrumental variable bias. F = [R2/(1-R2)] × K × [(N-K-1)], where K is the number of single-nucleotide polymorphisms, N is the GWAS-exposed sample size, and R2 is the % of variation in the exposure database that can be explained by single-nucleotide polymorphisms. (R2 = 2 × (1-MAF) × MAF × β/SD), where MAF is the minor allele frequency, which is comparable to the allele frequency effect; β is the allele’s effect value (beta, β); SD = sx × √N; and sx (standard error) is the standard error of β.

Outliers for horizontal pleiotropy were tested and calibrated using MR Pleiotropy RESidual Sum and Outlier (PRESSO) prior to the MR analysis. To guarantee the validity of MR evaluation, the outliers in the instrumental variables are eliminated using the model’s outlier-corrected approach, and the effect evaluation is carried out following the correction of the horizontal pleiotropy. These carefully chosen single-nucleotide polymorphisms were then employed as instrumental factors in the following two-sample MR analyses.

### MR analysis

The IVW technique, weighted median (WM) method, weighted median estimator (SM) method, and simple median (SM) method, were all employed in this study’s two-sample MR technique and MR–Egger regression in order to confirm the causal relationships between genetic variants in statins and hip, knee, and osteoarthritis. In this analysis, the IVW method, which is based on the aggregated data on genotypes, was used as the main method to combine Wald estimates obtained through meta-analysis for every single-nucleotide polymorphism, yielding the total estimate, and the weighted regression slope of the resultant effect to the susceptibility effect represented the outcome estimate (the intercept limit was zero). WME was used to improve estimates of IVW and then obtain a consistent estimate of causality, with WME allowing for the presence of invalid SNPs and using more than half of valid SNPs for causal estimates. WM weights the causal effect value of each SNP pair with the number of SNPs in each cluster, and the returned result is a provisional estimate with the maximum SNP number weight. SM classifies SNPs according to causal effects and divides similar values into clusters, using clusters with the highest number of SNPs to evaluate the estimated causal effects. With the help of MR–Egger regression, all single-nucleotide polymorphisms are pleiotropic, which can identify horizontal heterogeneity by intercept testing and give estimates after pleiotropic correction.

### Observational index

The observational indices are as follows: (1) causal association between statins and osteoarthritis. (2) Causal association of statins with site-specific knee osteoarthritis and hip osteoarthritis. (3) Sensitivity analysis verified the reliability of the results.

### Statistical analysis

Cochran’s Q test was employed to evaluate heterogeneity in the effects of statin-related single-nucleotide polymorphisms on outcomes of knee and hip osteoarthritis. When *p* < 0.05, it is considered that the single-nucleotide polymorphisms are heterogeneous, and then, the random-effects model of IVW is used for causal inference. Finally, in order to find out the potential pleiotropy, the MR–Egger intercept test was carried out, and the *p*-value of the regression intercept was used to estimate the extent of the horizontal pleiotropy effect. When *P* > 0.05, it meant that a lack of horizontal pleiotropy existed. Finally, the leave-one-out sensitivity test is carried out to evaluate whether the combined IVW estimate is affected by any single-nucleotide polymorphism. The results of MR evaluated by other instrumental variables are very different from the overall results after one instrumental variable is removed. This indicates that the MR results are susceptible to this tool variable. The results were expressed as an odds ratio (OR) and a 95% confidence interval (95%CI), and P < 0.05 was regarded as statistically significant. Using R language (version 4.3.0) software packages such as “two-sample MR,” “MR-PRESSO,” and “mr.raps,” all data analyses were carried out.

## Results

### Instrumental variables

According to the screening criteria for instrumental variables in this study, a total of 128 closely related single-nucleotide polymorphisms without linkage imbalance were first screened from the statin exposure dataset. After several screenings, 115 single-nucleotide polymorphisms were obtained from the osteoarthritis outcome dataset. The PhenoScanner database was used to search related phenotypes, and one single-nucleotide polymorphism (rs1415991), whose corresponding phenotype was associated with osteoarthritis, was deleted, and the palindromic single-nucleotide polymorphism (n = 23) was deleted. At the same time, one outlier (rs117814720) was eliminated after MR-PRESSO analysis, and the distribution range of F-statistic corresponding to a single-nucleotide polymorphism was 22.10–257.72, representing that there was a lack of weak instrumental variable in MR analysis. Finally, 90 single-nucleotide polymorphisms were included as instrumental variables to assess the relationship between statins and osteoarthritis.

Overall, 128 single-nucleotide polymorphisms were obtained from the knee osteoarthritis outcome dataset. Two single-nucleotide polymorphisms (rs78087637 and rs998584), whose corresponding phenotypes were associated with knee osteoarthritis, were excluded, and palindromic single-nucleotide polymorphisms (n = 22) were deleted. At the same time, three outliers (rs12112877, rs115478735, and rs9849171) were eliminated after MR-PRESSO analysis. The distribution range of F-statistic corresponding to a single-nucleotide polymorphism was 25.50–366.85, representing that there was a lack of weak instrumental variables in MR analysis. Finally, 101 single-nucleotide polymorphisms were examined in order to evaluate the correlation between statins and osteoarthritis in the knee.

Overall, 128 single-nucleotide polymorphisms were obtained from the hip osteoarthritis outcome dataset. One single-nucleotide polymorphism (rs1415991), whose corresponding phenotype was associated with hip osteoarthritis, was excluded, and the palindromic single-nucleotide polymorphism (n = 23) was deleted. At the same time, three outliers (rs112108223, rs213484, and rs217181) were eliminated after MR-PRESSO analysis. The distribution range of F-statistic corresponding to a single-nucleotide polymorphism was 25.67.10–378.92, indicating that there was a lack of weak instrumental variables in MR analysis. Finally, 101 single-nucleotide polymorphisms were included as instrumental variables to assess the correlation between statins and hip osteoarthritis.

### Effects of statins on osteoarthritis disease

**FIGURE 1:**
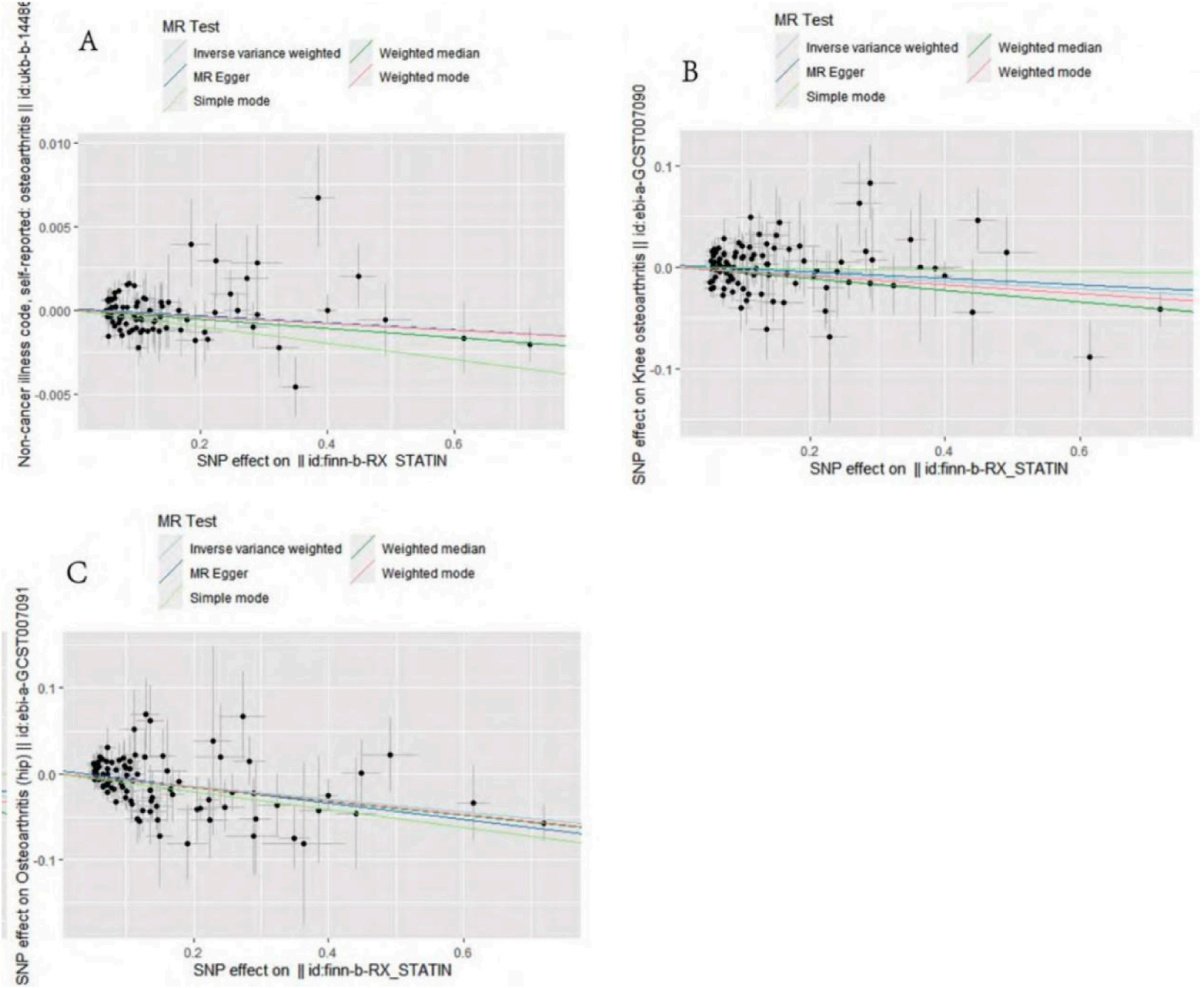
Mendelian randomization effect scatter plot of causal association. **(A)** Statins and osteoarthritis. **(B)** Statins and knee osteoarthritis. **(C)** Statins and hip osteoarthritis.

IVW analysis showed that genetically predicted statin was associated with osteoarthritis (OR = 0.998, 95%CI: 0.996–0.999, P = 0.01), and knee osteoarthritis (OR = 0.964, 95%CI: 0.940–0.989, P = 0.005) and hip osteoarthritis (OR = 0.928, 95%CI: 0.901–0.955, P = 4.28 × 10^−7^) may have a negative causal effect with statistical significance. Meanwhile, the four other analytical methods (MR–Egger, WM, WME, and SM) were applied to confirm the robustness of the results. Among them, the analysis results of WM, SM, and WME methods are similar to those of IVW. Although the MR–Egger analysis does not support the relationship between statins and osteoarthritis (OR = 0.997, 95%CI: 0.995–1.000, P = 0.089) OR knee osteoarthritis (OR = 0.968, 95%CI: there is a causal relationship between 0.930–1.008 and P = 0.121), the results of five analytical methods, IVW, MR–Egger, WM, WME, and SM show the same direction (OR values are all <1), as shown in Table 2. In addition, the causal estimation of the MR-PRESSO method before and after the outlier correction is consistent, and the scatter plot also shows that the regression line of the genetically predicted statin risk for osteoarthritis, knee osteoarthritis, and hip osteoarthritis is basically consistent, as shown in Figure 1. The above methods indicate the reliable MR analysis results.

**TABLE 2:** Mendelian randomization of risks of osteoarthritis, knee osteoarthritis, and hip osteoarthritis using statins.

Exposure	Outcome	Mendelian randomized analysis method	Number of ultimately included single-nucleotide polymorphisms	OR (95%CI)	P
Statins	Osteoarthritis	Inverse-variance weighting method	90	0.998 (0.996–0.999)	0.01
		Mendelian randomized Egger intercept	90	0.997 (0.995–1.000)	0.089
		Weighted median	90	0.997 (0.995–0.999)	0.009
		Weighted median estimator	90	0.997 (0.995–1.000)	0.058
		Simple median	90	0.995 (0.991–0.999)	0.023
	Knee osteoarthritis	Inverse-variance weighting method	101	0.964 (0.940–0.989)	0.005
		Mendelian randomized Egger intercept	101	0.968 (0.930–1.000)	0.121
		Weighted median	101	0.943 (0.912–0.975)	<0.001
		Weighted median estimator	101	0.957 (0.925–0.990)	0.014
		Simple median	101	0.992 (0.914–1.077)	0.85
	Hip osteoarthritis	Inverse-variance weighting method	101	0.928 (0.901–0.955)	<0.001
		Mendelian randomized Egger intercept	101	0.909 (0.869–0.951)	<0.001
		Weighted median	101	0.923 (0.882–0.965)	<0.001
		Weighted median estimator	101	0.923 (0.889–0.959)	<0.001
		Simple median	101	0.901 (0.833–0.975)	0.011

### Sensitivity analysis

**FIGURE 2:**
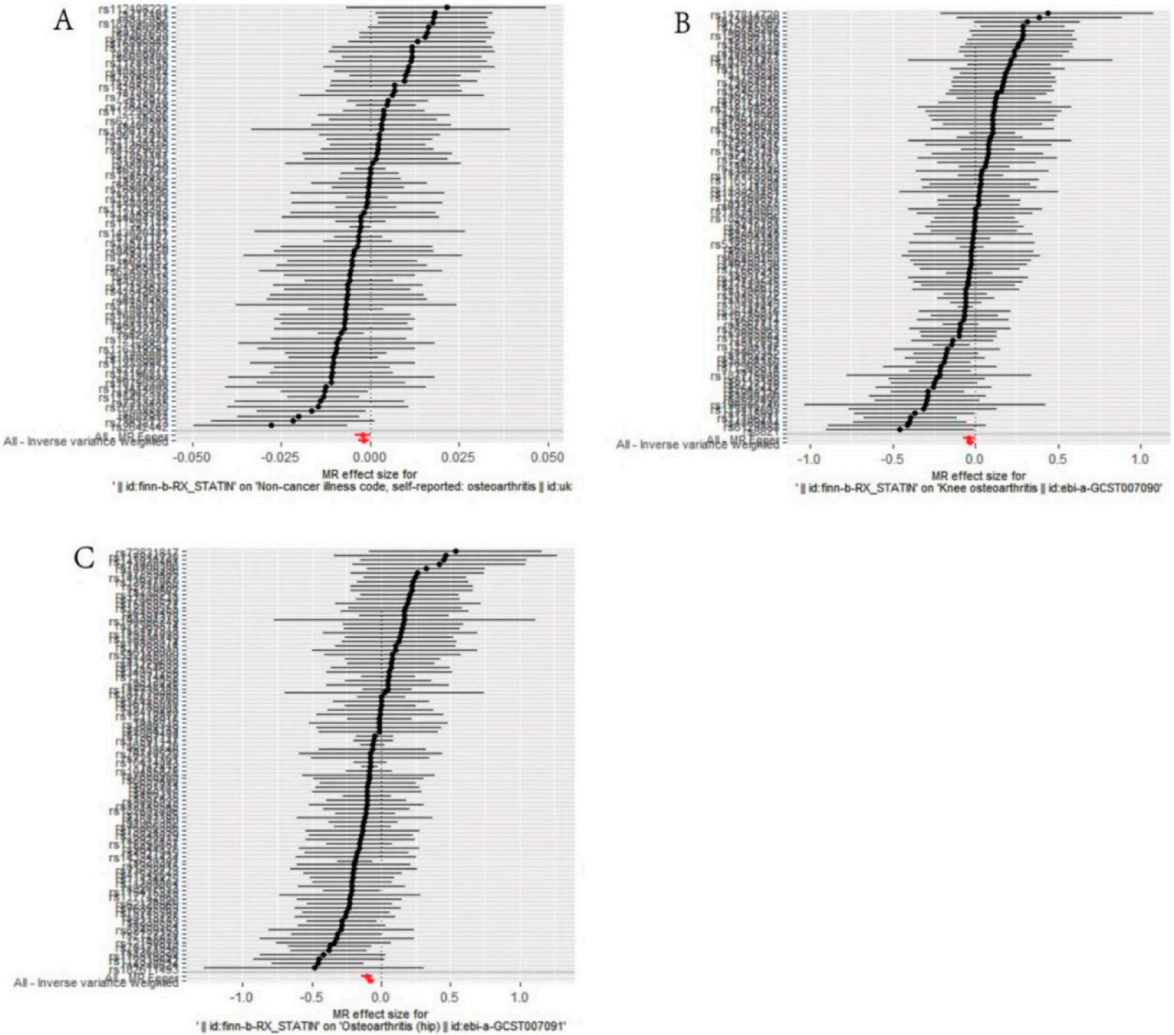
Mendelian randomization effect size forest plot of causal association. **(A)** Statins and osteoarthritis. **(B)** Statins and knee osteoarthritis. **(C)** Statins and hip osteoarthritis.

**FIGURE 3:**
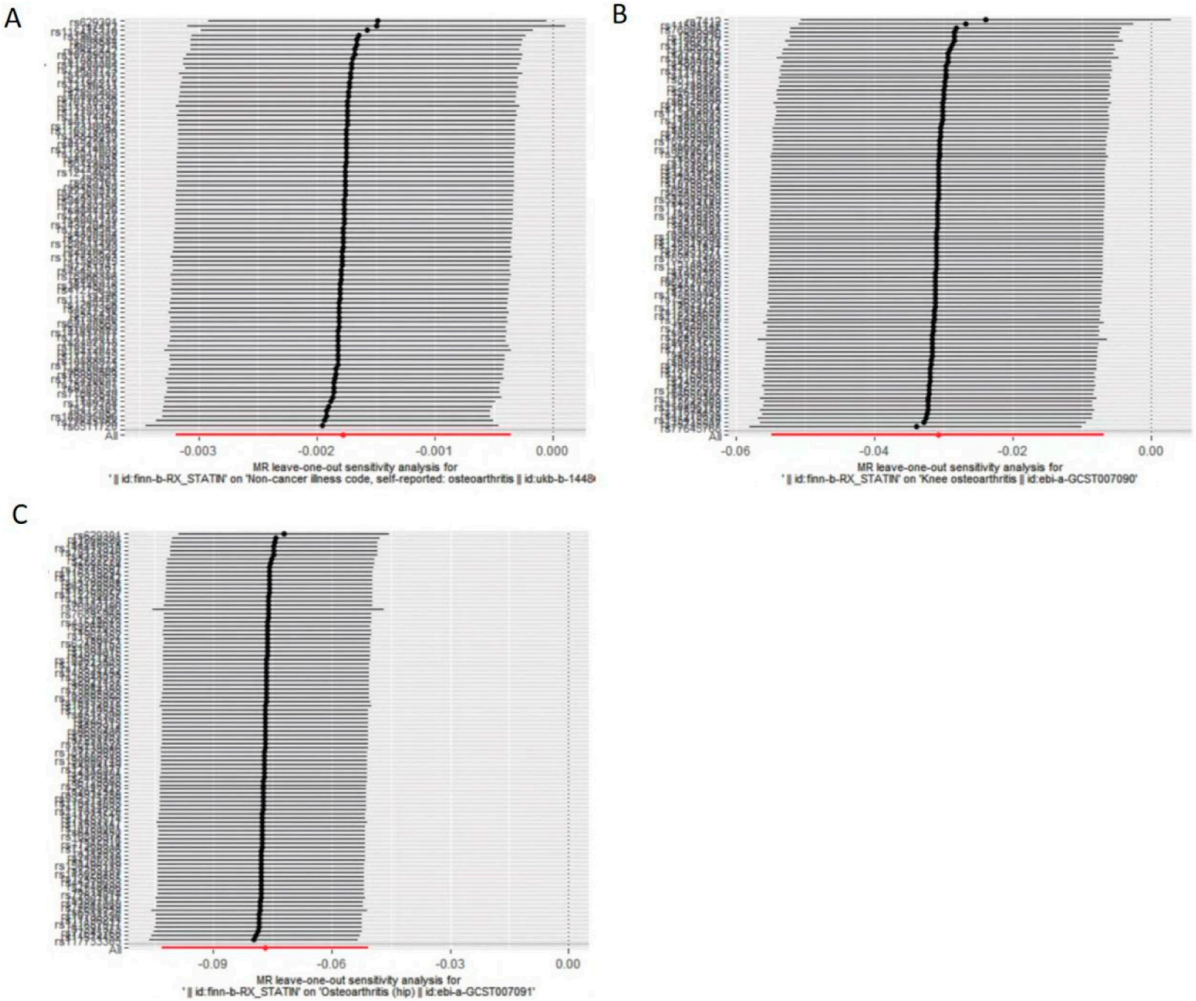
Evaluate whether a single variable drives correlation. **(A)**. Statins and osteoarthritis. **(B)** Statins and knee osteoarthritis. **(C)** Statins and hip osteoarthritis.

**FIGURE 4:**
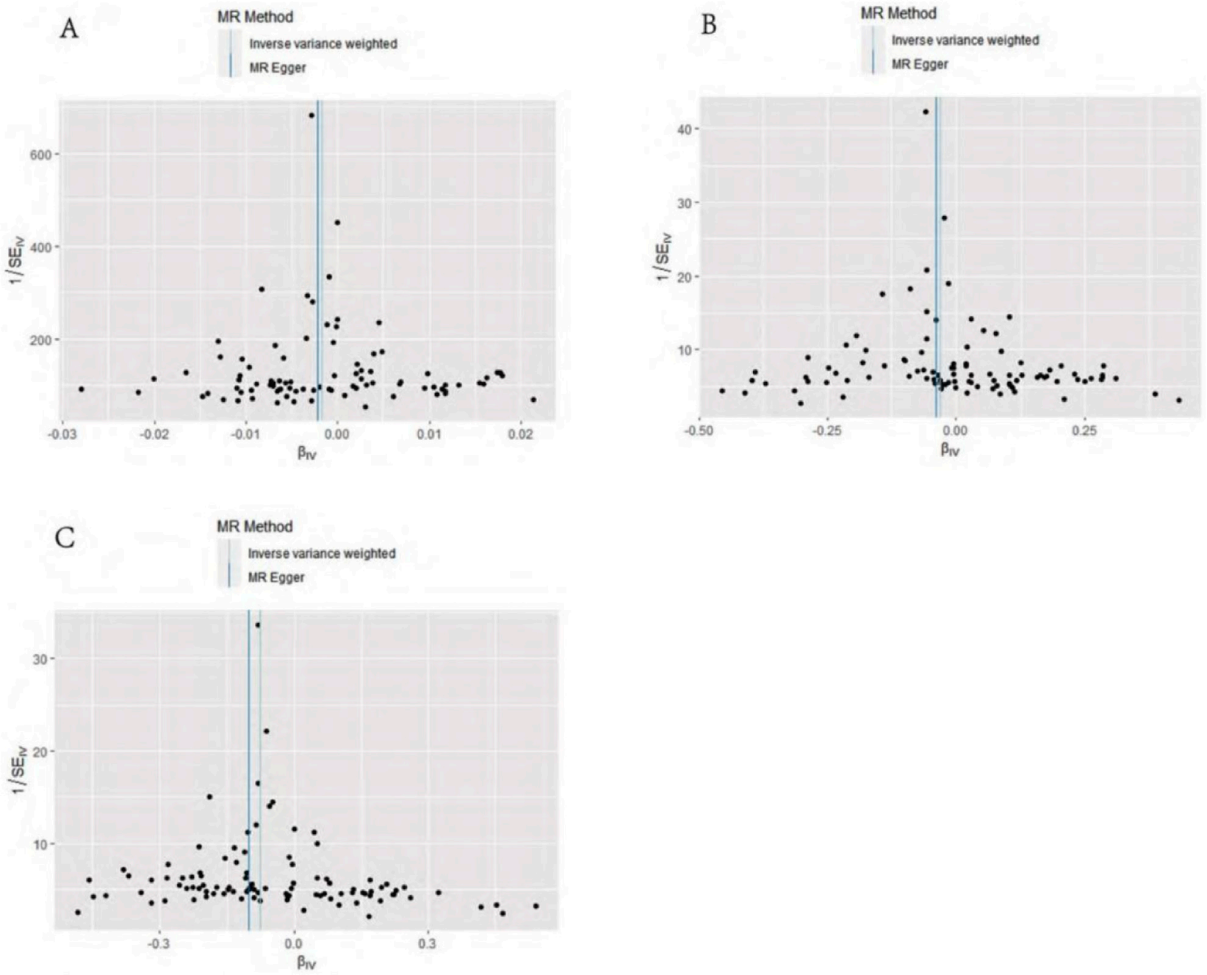
Funnel diagram of causation. **(A)** Statins and osteoarthritis. **(B)** Statins and knee osteoarthritis. **(C)** Statins and hip osteoarthritis.

MR–Egger intercept analysis did not detect potential horizontal pleiotropy (osteoarthritis: P = 0.658; knee osteoarthritis: P = 0.600; and hip osteoarthritis: P = 0.141), representing that instrumental variables did not remarkably affect outcomes by means other than exposure. In Cochran’s Q heterogeneity test, there was potential heterogeneity in knee osteoarthritis results (P = 0.015), while no significant heterogeneity was observed in single-nucleotide polymorphisms for osteoarthritis (P = 0.141) and hip osteoarthritis (P = 0.579), as shown in Table 3. Although heterogeneity was found, the IVW assay results demonstrated a causal relationship between statins and knee osteoarthritis (P = 0.005) and did not violate the horizontal pleiotropy test in core hypothesis 2. None of the individual single-nucleotide polymorphisms had an effect on the overall causal estimation, according to the leave-one test analysis. Figures 2, 3 display the leave-one test results and the forest map of the MR effect size of the causal relationship. To investigate the stability of the aforementioned findings further, the funnel plot displays a largely symmetrical causal effect distribution that is unaffected by potential causes, as shown in Figure 4.

**TABLE 3:** Multipotency and heterogeneity of statins in osteoarthritis, knee osteoarthritis, and hip osteoarthritis.

Exposure	Outcome	Pleiotropy test		Heterogeneity test	
		P (Mendelian randomization –Egger intercept)	P (Mendelian randomized PRESSO)	P (Mendelian randomized Egger Q)	P (inverse-variance weighting Q)
Statins	Osteoarthritis	0.658	0.115	0.128	0.141
	Knee osteoarthritis	0.6	0.015	0.013	0.015
	Hip osteoarthritis	0.141	0.404	0.614	0.579

## Discussion

Exploring the causal relationship between susceptibility factors and osteoarthritis disease is critical to the development of disease prevention and improved treatment strategies. With the rapid development of genetics and bioinformation technology, the measurement accuracy of genetic variation is constantly improved, which also significantly reduces the bias that may be caused by measurement errors in research, thus promoting the wide application of MR in medical research (Yan et al., 2021; Lin et al., 2022). Therefore, disease-related single-nucleotide polymorphisms were selected from the GWAS data as instrumental variables to replace exposure factors and evaluate the association between exposure and outcome. Since the data were not affected by the surrounding environment, a large number of sample data were collected, and MR could prevent possible confounding factors from having an impact on how accurate the association results are. Because of this, the correlation results are more trustworthy than those from observational research or even RCTs. (Wang et al., 2022; Funck-Brentano et al., 2019). The study—the first MR analysis of statins, osteoarthritis risks, and osteoarthritis in the knee and hip—showed that statins may have a significant causal association with a reduced risk of osteoarthritis, knee osteoarthritis, and hip osteoarthritis.

Several previous observational studies have explored the relationship between statins and osteoarthritis (Baker et al., 2011; Sarmanova et al., 2020). In terms of clinical treatment, a previous study found that long-term use of statins can reduce the incidence of osteoarthritis and can delay its progression (Mohajer et al., 2023). Another scholar conducted a retrospective study stratifying participants according to the presence or absence of Heberden’s nodes (HN) and found that statin use had a protective effect on the progression of osteoarthritis in participants with HN-positive presence (Haj-Mirzaian et al., 2019). Another experimental animal study found that intra-articular injection of statins into the controlled release system reduced joint inflammation in a rabbit model of osteoarthritis (Goto et al., 2017). However, another study has found that statin use may elevate the osteoarthritis risks, but this trend is not significant and has no adverse effect on the progression of osteoarthritis (Beattie et al., 2005). In addition, a study found that statins did not reduce the risk of hospitalization for hip and knee osteoarthritis. These studies suggest that there is still debate about whether statins can treat osteoarthritis (Wang et al., 2021; Jokar et al., 2020). Therefore, this MR study provides a theoretical basis for previous observational studies, and the detection of specific associated genes can predict the therapeutic effect. It also provides important new evidence for the rational use of statins in osteoarthritis patients, which is of great significance for disease prevention and the formulation of improved treatment strategies.

Various possible processes should be taken into consideration in order to establish the causal relationship between susceptibility and results derived from MR analysis. Even though the biological mechanism of genetic variation as an instrumental variable is frequently unclear, it can be understood on the basis of the biological mechanism, and such a causal relationship can appear reasonable and explainable (Ni et al., 2022). First, in osteoarthritis, interleukin 1 may be one of the most important pro-inflammatory cytokines, which, together with the tumor necrosis factor, can activate various harmful substances, including nitric oxide and matrix metalloproteinases, thereby causing metabolic stress-induced joint inflammation and osteoarthritis. In addition, reducing these compounds may help improve joint pain. In fact, interleukin 1 can induce the production of most proteases that cause cartilage destruction (Na et al., 2020). Interleukin 1 reduces local matrix synthesis and stimulates the release of other inflammatory transmitters, such as interleukin 6 and interleukin 8. In some cases, interleukin 6 can work synergistically with interleukin 1 to induce collagenase production (Tanaka et al., 2019). Second, statins reduce matrix metalloproteinase-13 produced by interleukin-1β-stimulated chondrocytes and exert immunomodulatory effects through T-cell activation and inhibition of interferon-γ-induced class II histocompatibility complex expression (Abeles and Pillinger, 2006). In addition, statins may also reduce the pain of osteoarthritis by inhibiting oxidative stress and protecting cartilage from degradation (Pathak et al., 2015). Third, the lipid-lowering effects of statins may play a role in preventing osteoarthritis (Gierman et al., 2014). Therefore, statins not only have the ability to lower blood cholesterol levels but also have anti-inflammatory and immunomodulatory properties, and combined with the results of this MR analysis, these findings provide insights into the role of statins in osteoarthritis prevention.

This study also has certain limitations: there is some heterogeneity in the analysis, which may result in heterogeneity due to the use of GWAS data and the inability to probe any potential non-linear relationships or classification effects that differ by age, health status, or sex. Although statins have shown potential benefits in the prevention of osteoarthritis, the specific treatment regimen and dosage still need to be determined by further research and clinical practice. The aggregated GWAS data included only people of European descent, the conclusions may not be fully representative of the overall population, and more research should be carried out in the future to validate the result applicability to other ethnicities.

## Conclusion

Taken together, the results of this study reveal a negative causal association between statins and osteoarthritis at the genetic level, and this causal association is also present in knee osteoarthritis and hip osteoarthritis. Statins have previously been used primarily to treat dyslipidemia and other cardiovascular risk factors, and some older patients are also more susceptible to osteoarthritis. This study provides potential evidence for the use of statins in the treatment of osteoarthritis, suggesting that improving statin initiation and adherence may be considered in the future clinical treatment of osteoarthritis. In addition, statins can also be combined with bone tissue engineering to enhance the biological properties of bone tissue engineering materials by increasing the multiplication and differentiation of bone cells, promoting the release of growth factors, improving the formation of extracellular matrix, and providing a new therapeutic approach for bone regeneration and repair.

## Data Availability

The datasets presented in this study can be found in online repositories. The names of the repository/repositories and accession number(s) can be found in the article/supplementary material.
